# Explaining the unmet information needs of family carers of people with dementia: a theoretical model of information behaviour

**DOI:** 10.1186/s12877-024-05626-6

**Published:** 2025-04-03

**Authors:** Chiara De Poli

**Affiliations:** 1 Department of Social Policy, London School of Economics and Political Science, Houghton Street, London, WC2A 2A UK; 2 Care Policy and Evaluation Centre, London School of Economics and Political Science, Houghton Street, London, UK

**Keywords:** Unpaid care, Family care, Dementia, Information behaviour, Information need, Unmet need

## Abstract

**Background:**

Family carers of people with dementia often report unmet information needs, despite policy emphasis on the provision of information as key to enabling good care and empowering carers in their role. Although the consequences of unmet information needs are detrimental to both the person being cared for and the carer, a systematic understanding of the causes of unmet information needs is lacking. To address this gap, this article presents a theoretical framework centred on the concept of information behaviour and integrating the information seeking and communication model, candidacy theory, and discrepancy theory. The framework maps information behaviour across six phases (from the identification of an information need to its satisfaction) and three levels (individual, service, system) at which explanatory factors may be observed.

**Methods:**

The framework was tested on data collected from 24 in-depth interviews and two focus groups with people with dementia and family carers of someone living with dementia in the North-East of England (UK). Data were analysed thematically to map the factors at play at each phase of the framework that might explain whether needs were met.

**Results:**

Unmet information needs are not always the result of a lack of information. Issues such as inadequate support for the user in identifying the need, problems in finding information, the timing of information provision, the amount of information provided, the credibility of the information source, and the relevance of the information (given care needs, preferences, personal, and family circumstances) can all contribute to unmet information needs. This work shows that meeting an information need ultimately requires progress through the different stages of information behaviour, each of which is influenced by the interplay of individual-, service-, and system-level factors, and depends on both users and providers.

**Conclusions:**

This work challenges the rational paradigm in health and care information, which assumes that more information will lead to better care, and contributes to a critical perspective on health and care information that reframes successful information behaviour as a set of complex activities that are relational, emotionally charged, contextually embedded, and require (and produce) situated knowledge.

**Trial registration:**

Not applicable.

**Supplementary Information:**

The online version contains supplementary material available at 10.1186/s12877-024-05626-6.

## Background

With ongoing population ageing, the number of people living with dementia is projected to increase and dementia will continue to be one of the major causes of disability and dependency among older people, internationally and in the UK [1, 2]. Meeting the escalating demand for dementia care will pose significant challenges to health and care systems, long term care in particular [3–6].

To mitigate these epidemiological, social, and financial challenges, ‘ageing in place’ has been internationally promoted to support people with dementia to live independently or with assistance (often from families and friends) in the community for as long as they wish, with the aim of postponing or avoiding the use of residential care [7, 8]. In the UK, ageing in place, which has been the overarching framework of dementia policies since 2010 [9, 10], has been implemented under increasingly challenging contextual conditions. Ongoing shortages in the care workforce and years of fiscal austerity have led to a contraction in publicly funded formal care provision and an expansion of the role of family carers [11, 12]. By filling the gaps left by formal services, family carers support older people to live in the community and de facto enable the ageing in place strategy. Among the various roles that family carers take on [13] is that of “health information managers” [14] who locate, keep track, negotiate access to, and act upon information about the condition, treatment, care, and support services.

The importance of information in enabling and supporting carers in their role is recognised in policy documents (e.g., the Carers Action Plan [15], the Prime Minister’s Challenge on Dementia [9], the NHS Long Term Plan [16]), yet extant research has shown that carers’ needs for information are widespread and frequently unmet [17–21]. Underlying causes are manifold. They include a lack of available information [20, 22, 23], poor alignment between the topics usually addressed by information materials and what family carers need or want to know [24, 25], difficulties in navigating different sources of information [13, 26], and over-reliance by care professionals on signposting to online information sources, despite carers’ preference to receive information in the context of an encounter with a professional [27]. Furthermore, information needs vary along the disease trajectory [28] and according to personal and family circumstances [23, 29, 30]. However, a standardised approach to information provision is often used: information is often given at predetermined points in the care pathway [31] or is not culturally tailored [32], which, as a result, may affect the satisfaction of information needs [33, 34].

An unmet information need can have substantial consequences for those living with dementia and their family carers. Lack of, or fragmented, information about local services may lead to delayed service use or prevent service use altogether [35, 36]. Family carers may miss opportunities to learn coping strategies, access support to balance caregiving and other responsibilities [37], improve their understanding of dementia symptoms and behavioural changes in the person with dementia [38], and engage in advance care planning [39]. Ultimately, the lack of information has detrimental consequences for physical and mental health and quality of life of both those living with dementia and their family carers, may lead to inappropriate use of some services or premature use of residential care, and may contribute to carer burden [21].

This article aims to shift the discussion from describing the information needs of family carers of people with dementia to understanding why and how their information needs are met. It builds on the established concept of information behaviour, defined as the “generation, acquisition, management, use and communication of information, and information seeking” [40], which is used as the organising construct of the conceptual framework presented in this article.

After presenting the conceptual framework of information behaviour, from need identification through to need satisfaction, this article describes an empirical application to data collected with in-depth interviews with family carers of people with dementia living in a local area in the North-East of England (UK).

Data collection was conducted as part of a local co-creation study in which I was involved as part of a multi-disciplinary research team between 2015 and 2021 [41]. An advisory group including a person living with dementia and a family carer, alongside academics with relevant experience and professionals from local commissioning organisations, providers, and third sector organisations, oversaw the study.

## Conceptual framework

### Theoretical underpinnings

The conceptual framework builds on and integrates three existing theories. The Information-Seeking and Communication Model (ISCM) describes information behaviour by integrating the perspective of the information users with the perspective of the information provider [42]. Given that the ISCM is descriptive in nature, it was complemented with two explanatory theories. The first is Candidacy theory which defines candidacy as the ways in which people’s eligibility for intervention (access) is jointly negotiated between individuals and services [43]. This work broadens the initial scope of application of access, extending it from access to care to access to information. The second is Discrepancy theory, an expectation-based approach to the evaluation of care satisfaction which explains why a need is perceived as ultimately satisfied [44, 45].

#### Information Seeking and Communication Model (ISCM)

Within the ISCM model. an information user may be an individual, group, or organisation that has information need, seeks information, or uses information to decide or to act. An information provider is an individual, group, or organisation who produces, supplies, or communicates information, or who facilitates or controls access to it.

Information actors operate within personal and environmental contexts. The personal context represents the individual level factors that may influence information behaviour. These include an information actor’s demographics, expertise (including knowledge, education, training, and experience), and psychological factors, such as personality and mental processes around self-perception and self-efficacy, cognitive dissonance or cognitive avoidance, ability to cope with stress when cognitively appraising a situation, and perception of risk (i.e., the impact that would result from giving up information-seeking, balanced against the anticipated reward from accessing information which eliminates feelings of ‘not knowing’).

The environmental context is that in which the information actor lives or works. It encompasses location, culture, social influences (e.g., friends), professional and organisational culture, role-related factors (e.g., objectives, tasks, time constraints), financial constraints, and technological access to support information behaviour (e.g., telephone, Internet).

Personal and contextual factors may influence the needs, wants, goals, and perceptions that motivate or inhibit information behaviour, both from the user’s end, by influencing their decision to seek information, and the provider’s end, by influencing their decisions on whether, what, and how to communicate.

An information user who decides to seek information might have several information sources available to them, which are assessed on the basis of utility and credibility. Utility refers to the perceived usefulness, relevance, timeliness, accessibility, and ease-of-use of information or of a source; credibility refers to the perceived trustworthiness, authority, reliability, and lack of bias [42]. If information users deem that the information found is useful and credible, they use it to make decisions and take action. Otherwise, they can dismiss it and engage in further information-seeking, or make decisions and take action (or decide to take no action) on the basis of their existing knowledge. Decisions, actions, and knowledge are outcomes of the information-seeking and communication between the information user and information provider.

#### Candidacy theory

Candidacy theory frames access to a service or intervention as the outcome of successful negotiation between users and providers. On the one hand, users articulate (or try to articulate) their individual candidacy claims, which are influenced by their personal, social, and cultural circumstances, as well as their understanding of the health and care system, their previous experience of using services or of encounters with professionals [46]. On the other hand, service providers assess users’ claims using their own professional expertise and judgement, alongside service operating criteria or system-level factors [43]. Candidacy is thus defined and redefined through situated, contingent, and dynamic interactions between individuals and professionals embedded in organisational and social contexts.

Candidacy theory offers a conceptualisation of access developed around seven constructs. *Identification* of candidacy refers to the prerequisite recognition that a need requires intervention. *Navigation* refers to the ease with which access can be navigated by potential users, both cognitively (e.g., being aware of the services on offer) and practically (e.g., being able to mobilise the resources, in terms of transport, finances, time required to access services). *Appearance* at services pinpoints the competencies that a potential user is expected to deploy to credibly articulate their needs and reasons for access. *Offer and Resistance* refers to the response of a potential user to the offer of accessing a service. Non-utilisation may be a consequence of a non-offer or the deliberate choice of resisting that offer [47]. *Permeability* of services refers to the degree of alignment between users and services, for example determined by eligibility criteria for a service or pragmatic considerations (opening hours, language). *Adjudications* refer to the judgements and decisions made by professionals with respect to a candidacy claim: this can be shaped by a range of factors, from the subjective perception of the professional about the appropriateness of the intervention for the possible user [43], to service operating conditions (e.g., in terms of resource availability) to public discourses around entitlement, for example of specific population groups (e.g., migrants). Lastly, *system-level conditions*, e.g., the availability and suitability of local resources, represent the local and contingent contextual factors that shape and influence candidacy production.

Each construct influences how one’s individual candidacy (i.e., the negotiated individual eligibility to access) is framed and managed. The first six constructs can be viewed as transition points at which a person’s candidacy for care must be negotiated, and the seventh captures the broader context in which negotiations take place [33].

#### Discrepancy theory

Discrepancy theory is an expectation-based approach to the evaluation of users’ satisfaction with care and services received. The theory sees satisfaction as the outcome of comparison between individual expectations about care or services and the users’ actual experience, as a proportion of individual expectations [45]. Subsequent theoretical refinements stemming from Donabedian’s quality of care framework helped define the content of expectations in terms of *structure* (e.g., facilities, personnel), *process* (e.g., competency and communication skills of care professionals), and *outcomes* (physical and psychological), to reflect the efficacy of the service and the extent to which it was perceived to benefit and address users’ needs [48].

### The integrated conceptual framework

**Fig. 1:**
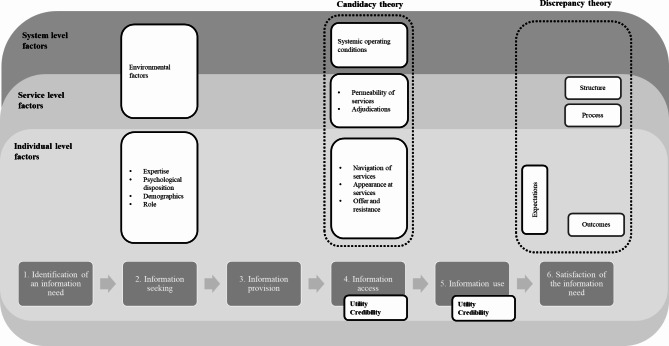
The conceptual framework

The six phases of the conceptual framework developed for this work largely replicate the phases of the information process described in the ISCM (Fig. 1):

1. Identification of an information need.
2. Information seeking.
3. Information provision.
4. Information access.
5. Information use.
6. Satisfaction of the information need.

Identification of a need for information is the first phase. An information need is defined as “a state or process started when one perceives that there is a gap between the information available to solve a problem and the actual solution of the problem” [49]. In this work information is broadly defined as facts, data, and knowledge about somebody or something which have been processed and organised with meaning and purpose [50].

An information need is the trigger and the driver of information-seeking [51]. In keeping with the ISCM, both environmental factors and personal characteristics shape the need identification phase. In the framework these are positioned as individual-, service-, and system-level factors, depending on the level at which they operate.

The second phase (information-seeking) encompasses the activities deployed to discover and access information resources that may help address the identified need, and thoughts and feelings associated with such activities. In the third phase (information provision), providers communicate with users either in response to an information-seeking process initiated by the user, or by anticipating an information need of the user. In the fourth phase (information access), the user decides whether and how to access the information made available to them or retrieved by them, influenced by its perceived utility and credibility. In the fifth phase (information use), information is assessed by the users and, if deemed relevant, is used, for example to make decisions or to take action. In the last phase, the information need that triggered the information behaviour is assessed against the information which has been accessed and used.

Candidacy theory is used to understand how the fourth phase (information access) unfolds and how this leads (or fails to lead) to the next phase of the framework where information is used. The seven constructs of candidacy are placed at the individual-, service-, and system-level of the framework.

Lastly, discrepancy theory is used in relation to the last two phases of the framework (information use and satisfaction of the information need) to explain why ultimately an information need is satisfied. Expectations and outcomes are positioned at the individual- level of the framework and structure and process on its outer levels.

## Methods

Twenty-four in-depth, semi-structured interviews and two focus groups (10 participants in total) with people living with dementia and family carers of people living with dementia were conducted as part of a co-creation initiative in the North-East of England (UK). The initiative, aimed at improving the local dementia care and support system, was organised in three phases. The interviews and focus groups were conducted in the first phase, which focused on exploring the lived experiences of people with dementia and their family carers as they navigated the local dementia care system. The improvement phase (phase 2) articulated and designed three interventions to address the priority issues identified in the diagnostic phase. Each intervention was then evaluated in phase 3.

Participants were invited to take part if they had received a diagnosis of dementia (any type, at any time prior to the interviews) or cared for somebody living with dementia, and were able to consent.

Recruitment of the purposeful sample of interviewees took place during 2017 using a maximum variation sampling strategy. The sampling framework included socio-demographic characteristics such as type of dementia, time from diagnosis, living arrangements, place of residence, socio-economic status, relationship between person with dementia and carer. Participants were identified through general practitioners, third-sector organisations, local commissioners, and care homes. Seventeen interviews were with family carers, two with a person living with dementia and five with a dyad (both the family carer and the person living with dementia when the latter needed support to take part) (Table 1). Two family carers who had recently experienced the death of a relative with dementia were included. Their experiences of dementia care were still relatively recent, having occurred within the previous twelve weeks, and were, therefore, deemed of interest for the study.

**Table 1:** Characteristics of interviewees

Id	Interviewee	Family carer			Person with dementia (PWD)					
		Age	Gender	Relation with person with dementia	Age	Gender	Years from diagnosis	Type of diagnosis	Disease severity	Geography
C1	Carer	68	Female	Daughter	93	Female	1	Mixed	Moderate/severe	Urban
C2*	Carer	54	Female	Daughter	86	Male	4	Alzheimer’s	Moderate/severe	Urban
C3	Carer	56	Male	Son	77	Female	5	Alzheimer’s	Moderate/severe	Urban
C4	Carer	69	Female	Partner	74	Male	2	Lewy body	Moderate	Urban
C5	Carer	69	Female	Spouse	76	Male	9	Alzheimer’s	Moderate	Urban
C6	Carer	57	Female	Daughter	81	Female	4	Alzheimer’s	Moderate	Rural
D1	Dyad				80	Female	4	Alzheimer’s		Rural
P1	Person living with dementia				72	Female	4	Mixed		Rural
C9	Carer	53	Female	Daughter-in-law	79	Female	5	Mixed	Mild/moderate	Urban
D2	Dyad				82	Male	1	Not known		Rural
C11	Carer	74	Female	Spouse	80	Male	8	Mixed	Severe	Rural
C12	Carer	68	Female	Spouse	77	Male	22	Vascular	Severe	Rural
P2	Person living with dementia				79	Male	1	Not known		Rural
C14	Carer	71	Female	Spouse	73	Male	9	Vascular	Severe	Rural
D3	Dyad				80	Female	1	Vascular		Rural
C16	Carer	59	Female	Spouse	66	Male	3	Lewy body	Mild/moderate	Urban
C17*	Carer	70	Female	Spouse	72	Male	NA	Vascular	Severe	Rural
C7	Carer	87	Male	Spouse	85	Female	6	Not known	Moderate	Urban
C8	Carer	69	Male	Spouse	65	Female	2	Alzheimer’s	Moderate	Rural
C10	Carer	68	Female	Spouse	81	Male	9	Mixed	Moderate	Rural
D4	Dyad				71	Female	2	Vascular		Rural
C13	Carer	57	Female	Daughter	86	Male	2	Mixed	Severe	Urban
C15	Carer	63	Female	Daughter	85	Female	2	Vascular	Moderate	Rural
D5	Dyad				86	Male	3	Vascular		Rural

*Person with dementia deceased

**Table 2:** Characteristics of focus group participants

		Family carer			Person with dementia (PWD)					
ID	Interviewee	Age	Gender	Relation with PWD	Age	Gender	Years from diagnosis	Type of diagnosis	Disease severity	Geography
FG01	Carer	64	Female	Spouse	66	Male	˂1 year	Alzheimer’s	NA	Rural
FG02	PWD				66	Male	˂1 year	Alzheimer’s	Mild	Rural
FG03	Carer	40	Female	Daughter	80	Female	2	Alzheimer’s	Mild/moderate	Rural
FG04	Carer	55	Male	Son	83	Female	˂1 year	Alzheimer’s	Mild	Urban
FG05	PWD				66	Male	4	Lewy body	Mild/moderate	Urban
FG06	Carer	59	Female	Spouse	66	Male	4	Lewy body	Moderate	Urban
FG07	Carer	76	Female	Spouse	80	Male	4	Mixed	Moderate	Urban
FG08	Carer	62	Female	Daughter	84	Male	NA	Alzheimer’s	Mild	Urban
FG09	PWD				84	Male	NA	Alzheimer’s	Mild/moderate	Urban
FG10	Carer	72	Female	Spouse	NA	Male	NA	NA	Moderate/severe	Urban

Interviews took place in the participant’s home and lasted on average one hour. The interviews aimed to explore the care experiences of people living with dementia and their family carers within the study site, focusing on individual care trajectories, patterns of service use, and met and unmet care needs. A semi-structured topic guide was used to provide flexibility in exploring the unique aspects of each participant’s care journey. Interviewees were invited to reflect on their experience and to think about the care and support services they accessed, used, refused, or stopped using, and the reasons for doing so. As part of the discussion, we explored their experience with these services, to understand, for example, whether they felt they were helpful, appropriate, provided at the right time or in the best place, and why. We also asked whether they were satisfied with the care and support they received, given their needs.

The focus groups were organised through local third-sector organisations. Participants were recruited using the same inclusion criteria. The focus groups were attended by 10 participants, seven family carers and three people living with dementia (Table 2). The focus groups lasted about 90 min and took place in a community space (November 2017) and on the premises of the hosting organisation made available for the event (February 2018).

Focus group participants were invited to discuss a selection of the themes that emerged from the analysis of the interviews, with the objective of understanding their experiences in relation to those themes and validating and expanding the interview data.

All participants were provided with detailed information sheets before taking part in the study, and written consent was obtained in accordance with the ethics approval granted. Special care was taken to ensure that the research processes, the consenting process in particular, were inclusive and responsive to participants’ needs. This included using plain language, breaking information into smaller, manageable segments, offering breaks during interviews and focus groups, and accommodating participants’ preferences, such as inviting or allowing a family member to attend with them the interview or the focus group. To support reflective practice, a research ethics log was maintained throughout the study to record ongoing reflections and considerations about implementing the approved ethics procedures in the field. Interviews and focus groups were audio-recorded with consent from participants, professionally transcribed, anonymised, and imported to NVivo12. Data were analysed thematically by type of care need and by type of interviewee (person with dementia, family carer), which allowed for a cross-sectional and granular view of the dataset, as previously described [47]. This stage of the analysis revealed a widespread and pervasive need for information as a key issue reported by family carers. The analysis then proceeded with the aim of explaining the observed variation in the satisfaction of family carers’ need for information. Hence, the initial codebook was developed inductively to (i) map the phases of the information behaviour and (ii) understand the factors at play in each phase that could explain whether the need was ultimately met.

Whilst the analysis proceeded, the codebook was iteratively refined and systematised using codes stemming from the theories underpinning the conceptual framework. For example, each of the seven constructs of candidacy theory were used to support the analysis of the information access phase.

The codebook at this point was piloted on a sample of transcripts. This helped understand whether the theory-informed codebook allowed capture of the specificities and nuances of the data, clarify the overall architecture of the framework, and further refine the codebook.

As the thematic analysis continued, a visual diagram was developed to represent the ramifications of the information process that could be identified from the empirical data (Supplementary material).

Through this process, it became apparent that some of the codes were relevant across phases and could not be attached to one specific phase only. For example, the code ‘Offer’ or ‘Adjudications’, that initially was placed only in relation to information access, played out already in the need identification phase. The codebook was further refined to account for these instances and was then applied to the full dataset.

## Results

Participants’ accounts highlighted the importance and pervasiveness of the need for information, which significantly influenced the experiences of care and support described by family carers and people with dementia. In the following sections I present an application of the conceptual framework to the empirical data collected from family carers.

### Identification of the information need

Participants’ accounts show that the need identification phase is far from being dichotomous (identification vs. non-identification). Instead, a typology can be constructed based on the extent to which needs could be identified by carers. This includes manifest need, emergent need, latent need, need identified by proxy, or need not identified.

Some participants explicitly identified a need for information, which may be defined as a manifest information need. They were able to recall that they had initiated the process of gathering information about dementia due to their limited knowledge on the subject. Hence, some decided to do dementia training

*“I did a level three course on dementia*,* which I passed*,* to try and help me understand Joseph …”[C5]*

Others proactively approached professionals to ask for information

*“I was ringing anybody and everybody just saying ‘I need to learn about dementia. I want to know how to do this’. It doesn’t come naturally…”[C6]*

Several carers researched independently, usually using the Internet

*“Whatever I asked*,* I got answers. They gave me some leaflets and*,* of course*,* you can always research yourself*,* can’t you? There’s so much information available and I am computer literate*,* so I looked [online].” [C15]*

In other instances, an information need was surfaced in serendipitous circumstances rather than through a structured, rational process. For example, carers often found out what they needed or wanted to know when talking with others in a similar position

*“A lot of the information that I’ve actually found out is from other people (…). They were the ones who said*,* ‘Do you know you can get council tax things?”. They knew more than anybody had actually offered to us.” [C4]*

For others, casual conversations with care professionals led to relevant pieces of information

*Carer: “I didn’t realise (…) that I could really get them (the carers) in for longer if I need to.”*

*Interviewer: “That was just because you were talking to the carer when she was here.”*

*Carer: “That’s right.” [C10]*

The emergent nature of the information need was also highlighted by some carers who felt it was intrinsic to their role

*“It would have been probably helpful to know what to expect*,* but I found it a learning curve really.” [C11]*

Some carers seem to have a more latent information need which was not explicitly identified because they could effectively draw on their pre-existing knowledge, as in the case of carers with previous caring experience

*Carer: “Polly has a little bracelet and if she gets into difficulty and I’m out*,* she just presses that*,* and they come through.”*

*Interviewer: “You found out about that through the information from [local third-sector organisation]?”*

*Carer: “No*,* we knew about that because we have a daughter who has MS and she’s on it …” [D4]*

Other carers used their pre-existing professional knowledge of some aspects of the English health and social care system to address their information need, as illustrated by a carer who had previously worked as a welfare benefits advisor

*Interviewer: “Had anybody given you any advice on that outside of your own knowledge?”*

*Carer: “No, no.” [D4]*

In other instances, the need was identified by a professional at a given point in time, not by the carers. The quote reported below is from a carer who described the time when she needed to arrange professional care for her mother

*“I thought that me mam had to pay for that [professional care] out of her attendance allowance and I thought we can’t afford it (…). And [the social worker] said ’No’ – that was one thing I didn’t know*,* and I hadn’t been told about – ‘No*,* (…) the attendance allowance is something separate (…)’. And I thought*,* well if I had known that maybe we would’ve done it earlier.” [C15]*

Lastly, in some cases the need was not identified by the carer or by professionals, as this quote illustrates

*“Oh*,* dear me. I don’t know*,* what is the next stage. Maybe I do need to speak to somebody from dementia*,* I know you [the interviewer] are from dementia services*,* aren’t you really? And you probably know…” [C1]*

### Information seeking

The data revealed three distinct types of information-seeking behaviour: active, opportunistic, and passive. Some participants did not display any information-seeking behaviour.

Those carers who were able to explicitly articulate an information need often sought out information in an active and deliberate way. This seemed to be influenced by several individual-level factors. First, the way in which some carers approached information-seeking was found to be contingent upon their personality. Some, more extrovert and sociable, enjoyed attending advice and information sessions, for example those organised by the local carers’ organisation. Others simply showed an assertive attitude that helped them seek out information

*Carer: “Everything I’ve been finding out is through listening, asking questions, pushing and keeping on.”*

*Interviewer: “Asking questions to whom?”*

*Carer: “Everybody and anybody who will listen to me basically until they get sick of me.” [C9]*

Second, IT literacy played a role, with tech-savvy carers able to access online resources, when needed

*“There was nothing pushed forward*,* and you would have to do a bit of research (…). The Internet helps a lot now*,* you can do your own research and*,* if you’re sensible*,* you can get where you need to be*,* but if you’re somebody who’s maybe not Internet literate*,* then you may have more of an issue.” [C3]*

Credibility of information sources, such as websites, was not explicitly appraised by participants, but it may have been inferred from the reputation of the organisation or their personal knowledge of the professional signposting to that specific resource, such as in this example

*“The doctor (…) said ‘Any information you need will be on website of [third-sector organisation].’ He told us where to go and what we needed…” [C3]*

Opportunistic information seeking behaviour was seen in those carers who relied on their personal or social network to acquire information serendipitously, often by word-of-mouth, as in the example of this carer who discovered the Council tax reduction scheme from other carers attending a local support group

*“One of the other carers said*,* ‘Did you know this?’ I said ‘No*,* I didn’t’.” [C16]*

In seeking information, some carers described casual use of social media. Often online sources were very accessible, requiring minimal effort to locate, and their credibility was not disputed. However, at times, this seemed to lead to mixed results

*“If you go on Facebook*,* there’s always adverts*,* and I think I must have just clicked that I would have these booklets. I’ll have a look at anything that might help but*,* sometimes*,* I think it can be confusing as well*,* you can overdo it.” [C4]*

Those carers for whom a professional assumed an information need because they were not able to articulate it themselves, had much more passive information-seeking behaviour, which seemed to be influenced by socio-demographic factors, such as age, or contending health issues

*“There was no information then… You just plugged on day-to-day-to-day*,* and when you’ve got health issues of yourself*,* you’re not really more aware of like*,* ‘Oh I can Google that and find out this and that and the other’.” [C12]*

Carers showing more passive behaviour seemed to rely consistently on a professional, such as a social worker or a dementia advisor, who then became their key source of information. Those who had not been allocated a named professional reported having a difficult time

*“If I’d just had a single point of contact*,* some sort of case worker… We’d been just left to pick our way through the system really*,* and to wade the crises as they happen*,* rather than somebody say*,* ‘Well this might happen and that might happen’”. [C2]*

Lastly, there were instances in which carers did not initiate any information-seeking or were not supported to do so. This seemed to be the case for carers who could not locate relevant information sources despite acknowledging, often explicitly, some degree of information need

*“I didn’t know where to go. I managed to get in touch with [third-sector organisation] and when she eventually got back [to me]*,* my mum was in here [care home]. They tell you all sorts of things*,* but not where do I go for help? What do I do next?” [C1]*

Equally, those carers who showed a more latent information need because they had previous experience of navigating the dementia care system, or the health and social care system more generally, either as users (as interviewee D4 whose daughter had MS) or in a professional capacity, did not engage in an explicit information-seeking phase. For them, need identification and information-seeking seemed to conflate into one single phase, as illustrated in the quote below, where the carer was a social worker

*“I haven’t been given information*,* but I haven’t looked for it because I know about such things because of me background.” [C16]*

### Provision of information

Information provision varied considerably in practice, even among those who actively sought information. For example, some received the information they expected only after explicitly asking professionals, as recalled by an interviewee

“*I can’t remember that I was given any written information. It was just a case of asking questions. (…) I’m sure it’s no fault*,* it’s just one of the things*,* they just don’t seem to offer the information unless you ask.” [C11]*

Other carers reported that they received verbal and written information from different sources, which they complemented with information they located independently

*“Whatever I wanted was there. Whatever I asked*,* I got answers. They gave me some leaflets and you can always research yourself.” [C15]*

Lastly, others could not locate the information they were looking for despite their attempts

*“I rang up and they just don’t seem to know.” [D2]*

### Information access

Information access can be explained by candidacy theory and its seven constructs, the first six representing transition points at which a person’s candidacy for information must be negotiated to gain access, and the seventh capturing the broader context in which candidacy claims are made [33]. It is worth noting that although candidacy aims to explain how access, as an independent phase, unfolds, its seven constructs, in combination, are at play throughout the phases of information behaviour that lead up to access.

Among those participants who did not acknowledge an information need, *offer* did take place if they were supported by a care professional who had recognised the information need and provided them with relevant information. Conversely, it did not take place when the information need went unrecognised also by professionals. Interestingly, a few carers whose information need was assumed by professionals and, hence, were offered information material or signposted towards information sources, turned down those offers (*resistance*), as in the extract below

*Interviewer 2: “Have you ever been given a document or a leaflet that has explained what to expect from dementia or what you can access?”*

*Carer: “They’ve given me the leaflets*,* but as soon as I start looking at them*,* I’m thinking ‘Oh God…’ I can’t be arsed to read all that.”*

*Interviewer 2: “Because it’s heavy?”*

*Carer: “Because it’s heavy. (…) it’s too much.” [C13]*

The category of *adjudications* was at play at the need identification stage for those carers who did not identify an information need independently. Their information need was identified for them by a professional

*“I was advised (…) by the CPN [Community Psychiatric Nurse] that I would be eligible for attendance allowance (…). Because*,* to be honest*,* even though I’m quite familiar with things that are going on*,* I had thought that that was a means-tested allowance*,* and it isn’t.” [C11]*

Examples of the category of *appearance* were provided by some carers who recognised their information need but somehow failed to initiate information-seeking behaviour. Often, they were not able to articulate their information need in concrete terms or were not able to identify the right source of information, in the form of written materials or a professional, for their specific information need

*“I didn’t know where to go*,* what to say.” [C1]*

Carers also highlighted the issues they experienced when trying to navigate the different sources of information (*navigation)*

*“I only look for help when I need it. If anything happens*,* I’ve got to start and where do I start? I’m not very good on the website myself*,* but I do go on and find out*,* but it’s where do I start from there?” [FG8]*

Carers’ accounts of fragmented and incomplete information materials and sources reflected the fragmentation of the dementia care system itself, involving different organisations across the health and social care systems. Such fragmentation affected *permeability*, i.e., the ease with which people could find and access the information they needed when needed. Many carers described situations in which a professional with experience of the local care system and knowledge of the individual or their family helped them find their way through the system by primarily providing accessible and timely information. This is illustrated in the exchange below, where the interviewer highlighted how a social worker provided information about social benefits, legal matters, and local services and arranged paid and respite care for the carer

*Interviewer “It sounds as if for you [the social worker] knew all these different things…”*

*Carer: “She was a very knowledgeable lady.” [C8]*

According to candidacy theory, opportunities for repeated interactions between professionals and service users represent systemic operating conditions that influence candidacy [43]. Participants in the study could not provide evidence for such opportunities. Possibly as a consequence of the austerity climate, they highlighted high level of staff turnover. This was most likely a barrier to developing rapport between those living with dementia, their families, and their care workers and was not conducive to continuity of care

*Interviewer: “Were they [CPNs] coming to see you at home to see how she [referring to the wife] was doing?”*

*Carer: “Yeah*,* but they changed… I think there were two or three within a matter of weeks” [C7]*

Local practices around carer’s assessments affected the local production of candidacy as well. Although carer’s assessments represented a formal opportunity for discussing carer’s needs and information signposting, some participants reported negative experiences

*Interviewer: “Have you been offered at some point a carer’s assessment?”*

*Carer: “Yes, that’s a sheer waste of time”. [C9]*

### Information use

In most cases the use of information followed information access, with carers acting upon on the information they accessed or were offered

*Carer: “We got the medication from the neurologist and*,* at that point*,* she gave us some information about Lewy bodies dementia and lists of websites I could look at.”*

*Interviewer: “Have you acted upon it?”*

*Carer: “Yes*,* I’ve read everything I can. (…) I still do*,* I still look on the Lewy bodies dementia website.” [C16]*

In other instances, carers did not use the information they were offered

*“There was a woman who came here a couple of times*,* and she points out these things to you*,* make sure you’re aware that they’re there. Which I did have the information*,* but I didn’t do a lot about it.” [D1]*

### Satisfaction of the information need

When participants were satisfied with the information they had received or found, their information need was met, which translated into practical or psychological benefits, and often both. For example, participants described how some pieces of information they received were useful, or gave them reassurance, made them feel in control of their situation, or made a difference, as observed by a carer with substantial visual impairment who was providing support to his wife living with dementia

*“It was through them [the local carers’ association] that made the difference.” [C12]*

In other cases, participants were dissatisfied with the information available and described how their information need was not met

*“We should have more information when we were first told about dementia*,* the different medications you can get*,* and different [support] groups*,* what there is available. (…) Because you’re left high and dry.” [C10]*

In many instances, satisfaction of the information need was the pre-requisite for the satisfaction of other needs, as the quotes below illustrate. The first one refers to the experience of a couple. The woman was diagnosed with dementia and her carer husband also had memory problems and other health issues. Despite their complex circumstances, they recalled how they were left to their own devices for about six months during which they had no support, or information about available support options

*“They [social services] come and do an assessment. But (…) no one tells you there’s that support out there. (…) And I think the six months we went [without going out]*,* no one told me nothing (…).” [C8]*

The second quote refers to the experience of a son working full time and providing care for his mother with advanced dementia. In his interview he recalled how he did not know that they could access respite care until the social worker casually offered it, two years after the mother was diagnosed and eight weeks before she moved in a care home

*“I would think more availability for respite… getting to know about that earlier would have helped.” [C3]*

In keeping with discrepancy theory, satisfaction of an information need can be explained as the outcome of a comparison between participants’ actual experiences of information seeking and use against their expectations. Several participants described how they would have expected to be supported in their information behaviour, as in the quote below

*“It’s almost as if there needs to be a checklist somewhere that your loved one is diagnosed with dementia… Right*,* this is what you need to implement. You need a CPN [Community Psychiatric Nurse]*,* and you need a social worker*,* and you need to know about what benefits you can claim*,* you need to know where to get home-help. Almost like a checklist*,* and okay if you don’t want to access those services*,* but just to know that they’re available. You know these are the charities that you could contact*,* did you know about the council tax*,* little things like that.”[C2]*

This interviewee emphasized that information should be provided in a timely manner (e.g., at the point of diagnosis), in a concise and accessible format (such as a checklist), should be relevant to both the person diagnosed with dementia and their family, and should be comprehensive, regardless of the organisations or providers to which the information may refer. Their experience of information seeking, access, and use had had none of these features.

Using discrepancy theory at a more granular level, participants articulated expectations in relation to *Structure* and *Process. Structure* encompasses the organisational arrangements of the local dementia care system. In relation to this, participants highlighted that they lacked a single information source they could reliably access when looking for information

*“I must admit… one of my problems has been getting anybody to provide me with an adequate source*,* where I could go in once*,* rather than have to go to all the different sections.” [C7]*

Their experience of a fragmented information system mirrored the organisational fragmentation of dementia care, with providers usually (but not always) offering information only about the services for which their organisation was responsible. Another related issue was the lack of a named care professional designated to offer comprehensive, person-centred support tailored to individual circumstances, and to provide care continuity.

*Process* relates to the soft skills of professionals supporting information behaviour of carers. Carers identified the importance of professionals showing empathy when providing people with information. This idea was often encapsulated in the idea of professionals taking the time to ‘sitting down’ or ‘holding hands’ when providing carers with the information they needed

*“If somebody could sit you down and say at the start of your journey*,* as they want to use these days… ‘This is the information that we have on it… It affects everybody differently*,* but you will be able to have support with this*,* that*,* and the other…” [C4]*

Professionals were also expected to be mindful of individuals’ readiness to engage in information behaviour. Some participants were not ready to seek or receive information and, hence, showed resistance when offered information or engaged only passively in information-seeking. Other participants would have been ready to receive information at an earlier stage. Had this occurred, they would have been better positioned to anticipate decisions and plan for the future

*Interviewer: “Do you think you’d have been interested in those [i.e.*,* information sessions provided by a third-sector organisation] if they’d’ve been helpful to you?”*

*Carer: “Yes*,* certainly because*,* I will sit down and think ahead all the time. I see what’s happening with her and then I see the latest problem*,* so I take steps all the way to try and be in front of that situation.” [D4]*

Lastly, professionals were also expected to provide at the *right* time the *right* amount of information. Some carers described how they received a large amount of information which was provided all at once (usually at the time of diagnosis) without due consideration for their individual willingness and readiness to process it

*“When we were getting the diagnosis… there was a lot of support services*,* came to see us and we felt very cared for*,* but a lot of information was thrown at us*,* we didn’t fully understand it*,* we didn’t know who to go to for what*,* and then suddenly it finished and that’s it.” [FG3]*

## Discussion

This article has delved into information behaviour in the context of dementia and family care and explored the factors that contribute to how and why information needs are fulfilled.

Empirically, this work drew on the experiences of family carers of people living with dementia among whom the need for information is not only prominent, but also a prerequisite to the satisfaction of other care needs, contributing to positive care outcomes and a positive caregiving experience [38, 52]. As such, this work offers three main contributions, the first of which is theoretical.

The framework maps the unfolding of information behaviour across six phases (from the identification of an information need to its satisfaction) and three levels (individual, service, system) at which relevant explanatory factors may play out. By design, the framework takes a need-based perspective: it starts from the identification of an information need and aims to understand whether such need is ultimately satisfied. The framework’s building blocks (i.e., phases, levels, and factors) help unpack users’ information behaviour, explore how the actual process of information need, seeking, and use occur, and ultimately explain the observed variation in satisfaction of an information need.

The framework integrates the ISCM with candidacy theory and discrepancy theory, hence overcoming the limitations of using each theory in isolation. Although the ISCM provides a unified description of information behaviour and highlights important factors in the information-seeking and communication process, it does not give a detailed representation of every aspect of information behaviour [53, 54]. Similarly, candidacy theory and discrepancy theory provide an in-depth understanding of specific phases of information behaviour, but can only partially explain why an information need is met or not. The integrative approach of the framework enhances the explanatory power of the individual theories.

By taking this approach, the framework challenges the rational information paradigm embedded in the health and care policy discourse. The rational approach to information assumes that the availability of more or good information will lead to good care and better outcomes in a somehow mechanistic way [55–58]. Information is expected to travel mainly unidirectionally from professionals to users, and information users carry responsibility for finding, using, and making sense of the information that is available.

This work instead contributes to a critical perspective on the information paradigm in health and social care by suggesting how activities encompassed under information behaviour are anything but mechanistic. Rather, they are intrinsically relational, often emotionally charged, contextually embedded, and require (and produce) situated knowledge [55, 56, 59]. In this vein, the framework establishes a link between information behaviour (as a set of tasks) and the concept of information work [57] which underscores how information-related activities involve the labour of ‘sifting through, interpreting, and dealing with the implications of the information one finds’ [14].

The second contribution of this work lies in its empirical nature. The framework serves as a heuristic tool for describing information behaviour across six phases and three levels. To start with, this work shows how identification of the need comes about. This may happen when people have a manifest need and can articulate it explicitly, when they have an emergent need, which is identified in a casual or serendipitous way, or when they have a latent need, which is not surfaced explicitly but can be addressed with their tacit or pre-existing knowledge. It may also happen when people who do not recognise their information need themselves are supported by a professional to do so. There also instances of unknown unknowns, in which an information need is not identified at all, when people are not able to identify it themselves and are not being supported to do so.

This work also shows how the identification of an information need is a complex process in relation to who recognises the need (e.g., the family carer, a professional), the level at which the need is recognised (from being manifest and explicitly identified to being ignored), and the variables that contribute to generate the need (e.g., role-related, psychological, cognitive). This work questions the generic role of carer as inevitably carrying an information need. This could be explained by the fact that the carer role itself is embedded in social and cultural norms and encompasses a wide range of aspect [60, 61], not all necessarily triggering an information need.

The study yields empirical evidence in support of theoretical models that suggest a link between cognitive and psychological variables and recognition of an information need [62]. It finds a positive interplay between need identification and individual self-efficacy, as the self-belief in one own capabilities to be able to influence events that affect their lives [63]. Personality, previous relevant experiences, and access to and confidence in leveraging relevant resources (including technology), seemed to contribute to the self-efficacy of some carers, who were able to verbalise their information need, tap into pre-existing knowledge, and mobilise new resources to help address the need.

Consistently with previous work [64], this work also finds a negative association between high level of stress and lack of coping strategies and identification of an information need. Avoidance was a common behavioural response among carers who seemed to experience high level of stress due to the intensity of their caring alongside other competing roles, the perception of multiple, possibly wide, knowledge gaps, and the uncertainty surrounding their situation. Some in this position were supported by professionals in retreating from avoidance and moving towards acknowledging an information need. Professionals helped them to regain a sense of agency by making sense of their circumstances and addressing their knowledge gaps. Others were not supported and were left to their own devices.

These cognitive and psychological factors contribute also to explain whether and how carers approached the information seeking phase, confirming established typologies of information seeking (active, opportunistic, passive) [65] and echoing the results of a review of the empirical literature [38].

Participants’ accounts also showed that the provision of information is an unstructured and fragmented phase which no service or professional seems to be responsible for, to the point where it might not happen. This seems to contradict the policy push towards making information widely available to support and empower carers in their roles. Whether and how information becomes available to them seems to depend on the resources that carers can deploy to find and access information rather than on what information providers make available.

Applying candidacy theory shows not only the complexity of the information access phase, but also how access comes about. Carers who were able to navigate the dementia care system, experienced favourable adjudications by professionals, were able to articulate their information needs (e.g., in the context of meaningful carer’s assessment), could access the information they needed. Carers who could not articulate their information need – failing on the *appearance* dimension – or were not able to navigate the information system in the information-seeking phase, and who could not rely on a professional helping them to do so (the *adjudication* and the *permeability* dimensions of candidacy) struggled to access the information they needed. These carers may be the ones at risk of falling through the cracks of complex care system and who would benefit most from trusting relationships and continuity of care. Some carers were offered support by a professional who assumed their information needs, but some put up a degree of *resistance* when the professional explicitly offered them information (favourable *adjudication*) or support with information-seeking (*navigation*, *permeability*).

Data show that there are instances where people do not use the information that they are provided with. This finding contributes to the body of literature [55–57] that disputes the positioning of good care as resulting from the availability of good information, which has been endorsed by the health and social care policy discourse in recent years.

This work also provides empirical evidence of the benefits of successful information behaviour, in terms of feeling in control, being reassured, being able to act, plan, and make decisions. Conversely, it also illustrates the consequences of unsuccessful information behaviour, in terms of stress and burden.

As a heuristic, the framework can be used to zoom in on any phase of information behaviour to understand how it unfolds, but can be used also to zoom out and capture information behaviour as a whole. When doing so, it shows that although the actual unfolding of information-seeking and communication processes is less linear and sequential than the visual representation of the conceptual framework suggests, the satisfaction of an information need ultimately requires progression across the different phases of the framework and the involvement of both users and providers. The data shows that an unmet information need does not always appear to be due to a lack of information per se. Indeed, this work highlights the multiple factors that may contribute to an unmet information need, from lack of adequate support in the need identification phase to issues in information retrieval, the timing of information provision, the amount of information provided, the credibility of the information source, and the relevance of the information available or offered, given care needs, preferences, personal, and family circumstances.

The application of the framework in the context of family care for people with dementia raises implications for both policy and practice, which are the final contribution of this work. Family carers may have an information need in relation to many topics and these topics may change over time, as a result of changes in needs, for example due to the progression of the disease, or to changes in personal and family circumstances [62]. In practice, carers may go through several cycles of information behaviour from the point when they start providing care and support to somebody living with dementia [66], with each iteration of the information behaviour potentially involving different sets of information providers and resources. Hence, interventions that support information behaviour under these circumstances are needed. Information resources which take a person-centred and relational approach, consider the information need as inherently subjective, are integrated across service providers (supported by guidance from a checklist as suggested by participants), and are offered as part of an encounter with a care professional, ideally in a context of care continuity from a dementia specialist, seem to be promising options in this regard.

The framework can also inform the design and evaluation of interventions to support information behaviour, such as the one described in [41]. It constitutes a programme theory that can be used prospectively, to articulate how an intervention is expected to work, or retrospectively, to evaluate how an intervention did work.

It is important to recognise strengths and weaknesses of this work. The framework is grounded in established theories and models in information and applied health research, which represents a strength of this work. Empirically, the study sample was heterogeneous with respect to the socio-demographic characteristics of the participants themselves, but it was ethnically homogeneous. As social and cultural norms can shape perceptions of dementia and family caregiving, interpretations of need, and expectations in relation to care [30, 67], future research should test empirically the framework with samples from ethnically diverse groups.

The empirical analysis focused on data collected from family carers of people with dementia, but the framework could help explain information behaviour among people living with dementia as well. It would also be interesting to explore information needs among people with lived experience of specific types of dementia (e.g., dementia with Lewy bodies [68] or young onset [69]. The framework could be used to explore the information need longitudinally, as the disease progresses, or at specific transition points (e.g., if a caring spouse dies). Future work could explore the relevance of the framework in a post COVID world where the reliance on information technologies has increased, the digital divide has widened, and IT literacy may play a greater role in explaining information behaviour than it did previously. Lastly, the framework could also be tested beyond dementia research.

## Conclusions

Although the provision of information has become central to dementia and family care policy in England over the last fifteen years, questions remain about whether the information that is provided meets the information needs of families caring for and supporting someone with dementia.

This article disputes the rational approach to information that has been adopted in policy and has been reflected in practice. Even when information providers (i.e., care professionals and organisations) provide more or ‘better’ information, this does not necessarily result in more informed, or more empowered information users, nor does it necessarily lead to improved care outcomes.

Using the concept of information behaviour, this article argues for a more critical approach to information which looks at how and by whom information is not only communicated, but also exchanged and generated. It shifts the focus from information provision and providers to recognise the active role of information users in a complex process where information is relationally constructed and embedded in context. In taking this approach, this article shows also how the concept of information behaviour has both analytical and policy affordances. Not only does it allow for a granular understanding of the variation in information needs that is observed in practice, but it should be at the heart of any policy and intervention in health and care information, in the context of dementia and family care, and beyond.

## Electronic supplementary material

Below is the link to the electronic supplementary material.

Supplementary Material 1

## Data Availability

The data that support the findings of this study are available from the corresponding author upon reasonable request.

## References

[1] Wittenberg R, Hu B, Jagger C, Kingston A, Knapp M, Comas-Herrera A, et al. Projections of care for older people with dementia in England: 2015 to 2040. Age Ageing. 2020;49:264–9. 10.1093/ageing/afz154. 31808792 10.1093/ageing/afz154 PMC7047814

[2] GBD 2019 Dementia Forecasting Collaborators. Estimation of the global prevalence of dementia in 2019 and forecasted prevalence in 2050: an analysis for the global burden of Disease Study 2019. Lancet Public Health. 2022;7:e105–25. 10.1016/S2468-2667(21)00249-8. 34998485 10.1016/S2468-2667(21)00249-8 PMC8810394

[3] Marešová P, Dolejs J, Mohelska H, Bryan LK. Cost of treatment and care for people with Alzheimer’s Disease: a Meta- analysis. Curr Alzheimer Res. 2020;16:1245–53. 10.2174/1567205017666200102144640. 10.2174/1567205017666200102144640 31894748

[4] Jönsson L, Tate A, Frisell O, Wimo A. The costs of dementia in Europe: an updated review and Meta-analysis. PharmacoEconomics. 2023;59–75. 10.1007/s40273-022-01212-z. 10.1007/s40273-022-01212-z PMC9813179 36376775

[5] Cantarero-Prieto D, Leon PL, Blazquez-Fernandez C, Juan PS, Cobo CS. The economic cost of dementia: a systematic review. Dement (London). 2020;19:2637–57. 10.1177/1471301219837776. 10.1177/1471301219837776 30909718

[6] Wittenberg R, Knapp M, Hu B, Comas-Herrera A, King D, Rehill A, et al. The costs of dementia in England. Int J Geriatr Psychiatry. 2019;34(gps5113). 10.1002/gps.5113. 10.1002/gps.5113 PMC6618309 30950106

[7] Pani-Harreman KE, Bours GJJW, Zander I, Kempen GIJM, Van Duren JMA. Definitions, key themes and aspects of ageing in place: a scoping review. Ageing Soc. 2020;1–34. 10.1017/S0144686X20000094.

[8] World Health Organization (WHO). Global action plan on the public health response to dementia 2017–2025. Geneva. 2017. Available: https://apps.who.int/iris/bitstream/handle/10665/259615/9789241513487-eng.pdf?sequence=1

[9] Department of Health. Prime Minister’s challenge on dementia 2020. London: Department of Health. 2015. Available: https://www.gov.uk/government/publications/prime-ministers-challenge-on-dementia-2020

[10] Department of Health. Living well with dementia: A National Dementia Strategy. London. 2009. Available: https://www.gov.uk/government/uploads/system/uploads/attachment_data/file/168220/dh_094051.pdf

[11] van Broese MI, De Boer A. Providing informal care in a changing society. Eur J Ageing. 2016;271–9. 10.1007/s10433-016-0370-7. 10.1007/s10433-016-0370-7 PMC4992501 27610055

[12] Wittenberg Y, Kwekkeboom R, Staaks J, Verhoeff A, de Boer A. Informal caregivers’ views on the division of responsibilities between themselves and professionals: a scoping review. Health Soc Care Community. 2018;e460–73. 10.1111/hsc.12529. 10.1111/hsc.12529 29250848

[13] Jamieson M, Grealish L, Brown JA, Draper B, Carers. The navigators of the maze of care for people with dementia—A qualitative study. Dementia. 2016;15:1112–23. 10.1177/1471301214554930. 25305279 10.1177/1471301214554930

[14] Harris R, Cyber-Burdens. Emerging imperatives in women’s unpaid care work. Gender, Health and Information Technology in Context. London: Palgrave Macmillan; 2009. pp. 72–87. 10.1057/9780230245396_5.

[15] Department of Health and Social Care (DHSC). Carers Action Plan 2018–2020 Supporting carers today. London, UK. 2018. Available: https://assets.publishing.service.gov.uk/government/uploads/system/uploads/attachment_data/file/713781/carers-action-plan-2018-2020.pdf

[16] NHS England. The NHS Long Term Plan. NHS England. 2019. Available: www.longtermplan.nhs.uk.

[17] Wancata J, Krautgartner M, Berner J, Alexandrowicz R, Unger A, Kaiser G, et al. The Carers’ needs Assessment for Dementia (CNA-D): development, validity and reliability. Int Psychogeriatr. 2005;17:393–406. 16252373 10.1017/s1041610205001699

[18] McCabe M, You E, Tatangelo G. Hearing their Voice: a systematic review of Dementia Family caregivers’ needs. Gerontologist. 2016;56:e70–88. 10.1093/geront/gnw078. 27102056 10.1093/geront/gnw078

[19] van der Roest HG, Meiland FJM, Comijs HC, Derksen E, Jansen APD, van Hout HPJ, et al. What do community-dwelling people with dementia need? A survey of those who are known to care and welfare services. Int Psychogeriatr. 2009;21:949. 10.1017/S1041610209990147. 19602305 10.1017/S1041610209990147

[20] Peterson K, Hahn H, Lee AJ, Madison CA, Atri A. In the Information Age, do dementia caregivers get the information they need? Semi-structured interviews to determine informal caregivers’ education needs, barriers, and preferences. BMC Geriatr. 2016;16:164. 10.1186/s12877-016-0338-7. 27662829 10.1186/s12877-016-0338-7 PMC5035467

[21] Whitlatch CJ, Orsulic-Jeras S. Meeting the Informational, Educational, and Psychosocial Support needs of persons living with dementia and their family caregivers. Gerontologist. Gerontological Society of America; 2018. pp. S58–73. 10.1093/geront/gnx162. 10.1093/geront/gnx162 29361068

[22] Vaingankar JA, Subramaniam M, Picco L, Eng GK, Shafie S, Sambasivam R, et al. Perceived unmet needs of informal caregivers of people with dementia in Singapore. Int Psychogeriatr. 2013;25:1605–19. 10.1017/S1041610213001051. 23845530 10.1017/S1041610213001051

[23] Greenwood N, Smith R. Barriers and facilitators for male carers in accessing formal and informal support: a systematic review. Maturitas. 2015;162–9. 10.1016/j.maturitas.2015.07.013. 10.1016/j.maturitas.2015.07.013 26271710

[24] Downs M, Clibbens R, Rae C, Cook A, Woods R. What do General practitioners tell people with dementia and their families about the Condition? Dementia. 2002;1:47–58. 10.1177/147130120200100106.

[25] Van Hout HP, Vernooij-Dassen MJ, Jansen DA, Stalman WA. Do general practitioners disclose correct information to their patients suspected of dementia and their caregivers? A prospective observational study. Aging Ment Health. 2006;10:151–5. 10.1080/13607860500310468. 16517490 10.1080/13607860500310468

[26] Morrisby C, Joosten A, Ciccarelli M. Do services meet the needs of people with dementia and carers living in the community? A scoping review of the international literature. Int Psychogeriatr. 2018;30:5–14. 10.1017/S1041610217001491. 28784193 10.1017/S1041610217001491

[27] Allen F, Cain R, Meyer C. Seeking relational information sources in the digital age: a study into information source preferences amongst family and friends of those with dementia. Dementia. 2018. 10.1177/1471301218786568. 29999410 10.1177/1471301218786568

[28] Tuijt R, Rees J, Frost R, Wilcock J, Manthorpe J, Rait G, et al. Exploring how triads of people living with dementia, carers and health care professionals function in dementia health care: a systematic qualitative review and thematic synthesis. Dementia. 2020;1471301220915068. 10.1177/1471301220915068. 10.1177/1471301220915068 PMC8047709 32212862

[29] Innes A, Morgan D, Kostineuk J. Dementia care in rural and remote settings: a systematic review of informal/family caregiving. Maturitas. 2011;68:34–46. 10.1016/J.MATURITAS.2010.10.002. 21093996 10.1016/j.maturitas.2010.10.002

[30] Greenwood N, Habibi R, Smith R, Manthorpe J. Barriers to access and minority ethnic carers’ satisfaction with social care services in the community: a systematic review of qualitative and quantitative literature. Health Soc Care Community. 2015;23:64–78. 10.1111/HSC.12116. 25135207 10.1111/hsc.12116 PMC4283974

[31] Wackerbarth SB, Johnson MMS. Essential information and support needs of family caregivers. Patient Educ Couns. 2002;47:95–100. 10.1016/S0738-3991(01)00194-X. 12191532 10.1016/s0738-3991(01)00194-x

[32] Kenning C, Daker-White G, Blakemore A, Panagioti M, Waheed W. Barriers and facilitators in accessing dementia care by ethnic minority groups: a meta-synthesis of qualitative studies. BMC Psychiatry. 2017;17:316. 10.1186/s12888-017-1474-0. 28854922 10.1186/s12888-017-1474-0 PMC5577676

[33] Koehn S. Negotiating candidacy: ethnic minority seniors’ access to care. Ageing Soc. 2009;29:585–608. 10.1017/S0144686X08007952. 23814327 10.1017/S0144686X08007952 PMC3693980

[34] Kelly F, Innes A. Facilitating independence: the benefits of a post-diagnostic support project for people with dementia. Dementia. 2016;15:162–80. 10.1177/1471301214520780. 24535818 10.1177/1471301214520780

[35] Bieber A, Nguyen N, Meyer G, Stephan A. Influences on the access to and use of formal community care by people with dementia and their informal caregivers: A scoping review. BMC Health Services Research. BioMed Central Ltd.; 2019. pp. 1–21. 10.1186/s12913-018-3825-z 10.1186/s12913-018-3825-z PMC6359781 30709345

[36] Stephan A, Bieber A, Hopper L, Joyce R, Irving K, Zanetti O, et al. Barriers and facilitators to the access to and use of formal dementia care: findings of a focus group study with people with dementia, informal carers and health and social care professionals in eight European countries. BMC Geriatr. 2018;18:131. 10.1186/s12877-018-0816-1. 29866102 10.1186/s12877-018-0816-1 PMC5987478

[37] Mastel-Smith B, Stanley-Hermanns M. It’s like we’re grasping at anything: caregivers’ education needs and preferred learning methods. Qual Health Res. 2012;22:1007–15. 10.1177/1049732312443739. 22645226 10.1177/1049732312443739

[38] Soong A, Au ST, Kyaw BM, Theng YL, Tudor Car L. Information needs and information seeking behaviour of people with dementia and their non-professional caregivers: a scoping review. BMC Geriatr. 2020;20. 10.1186/S12877-020-1454-Y. 10.1186/s12877-020-1454-y PMC7023704 32059648

[39] Dening KH, Jones L, Sampson EL. Advance care planning for people with dementia: a review. Int Psychogeriatr. 2011;23:1535–51. 10.1017/S1041610211001608. 21867597 10.1017/S1041610211001608

[40] Ingwersen P, Järvelin K. The turn: integration of information seeking and Retrieval in Context. Springer Netherlands; 2005.

[41] De Poli C, Oyebode JR, Binns C, Glover R, Airoldi M. Effectiveness–implementation hybrid type 2 study evaluating an intervention to support ‘information work’ in dementia care: an implementation study protocol. BMJ Open. 2020;10:e038397. 10.1136/bmjopen-2020-038397. 33293389 10.1136/bmjopen-2020-038397 PMC7725094

[42] Robson A, Robinson L. Building on models of information behaviour: linking information seeking and communication. J Doc. 2013;69:169–93. 10.1108/00220411311300039.

[43] Dixon-Woods M, Cavers D, Agarwal S, Annandale E, Arthur A, Harvey J, et al. Conducting a critical interpretive synthesis of the literature on access to healthcare by vulnerable groups. BMC Med Res Methodol. 2006;6:1–13. 10.1186/1471-2288-6-35. 16872487 10.1186/1471-2288-6-35 PMC1559637

[44] Pascoe GC. Patient satisfaction in primary health care: a literature review and analysis. Eval Program Plann. 1983;6:185–210. 10299618 10.1016/0149-7189(83)90002-2

[45] Williams B. Patient satisfaction: a valid concept? Soc Sci Med. 1994;38:509–16. 10.1016/0277-9536(94)90247-x. 8184314 10.1016/0277-9536(94)90247-x

[46] Mackenzie M, Conway E, Hastings A, Munro M, O’Donnell C. Is ‘Candidacy’ a useful Concept for understanding journeys through Public Services? A critical interpretive literature synthesis. Soc Policy Adm. 2013;47:806–25. 10.1111/j.1467-9515.2012.00864.x.

[47] De Poli C, Oyebode J, Airoldi M, Glover R. A need-based, multi-level, cross-sectoral framework to explain variations in satisfaction of care needs among people living with dementia. BMC Health Serv Res. 2020;20:657. 10.1186/s12913-020-05416-x. 32669104 10.1186/s12913-020-05416-x PMC7364635

[48] Donabedian A. Evaluating the quality of Medical Care. Milbank Mem Fund Q. 2005;83:166–203. 5338568

[49] Miranda S, Tarapanoff K. Information needs and information competencies: a case study of the off-site supervision of financial institutions in Brazil. Inform Res. 2008;13.

[50] Rowley J. The wisdom hierarchy: representations of the DIKW hierarchy. J Inf Sci. 2007;33:163–80. 10.1177/0165551506070706.

[51] Savolainen R. Information need as trigger and driver of information seeking: a conceptual analysis. Aslib J Inform Manage. 2017;69:2–21. 10.1108/AJIM-08-2016-0139.

[52] Kinchin I, Edwards L, Adrion E, Chen Y, Ashour A, Leroi I, et al. Care partner needs of people with neurodegenerative disorders: what are the needs, and how well do the current assessment tools capture these needs? A systematic meta-review. Int J Geriatr Psychiatry. 2022. 10.1002/gps.5764. [cited 4 Dec 2023]. 35665539 10.1002/gps.5764 PMC9328373

[53] Robson A, Robinson L. The information seeking and communication model: a study of its practical application in healthcare. J Doc. 2015;71:1043–69. 10.1108/JD-01-2015-0023.

[54] Savolainen R. Conceptual growth in integrated models for information behaviour. J Doc. 2016;72:648–73. 10.1108/JDOC-09-2015-0114.

[55] Barnes M, Henwood F. Inform with care: Ethics and information in care for people with dementia. Ethics Soc Welf. 2015;9:147–63. 10.1080/17496535.2014.969753.

[56] Barnes M, Henwood F, Smith N. Information and care: a relational approach. Dementia. 2016;15:510–25. 10.1177/1471301214527750. 24662501 10.1177/1471301214527750

[57] Dalmer NK. Add info and stir: an institutional ethnographic scoping review of family care-givers’ information work. Ageing Soc. 2020;40:663–89. 10.1017/S0144686X18001125.

[58] Henwood F, Harris R, Spoel P. Informing health? Negotiating the logics of choice and care in everyday practices of ‘healthy living’. Soc Sci Med. 2011;72:2026–32. 10.1016/J.SOCSCIMED.2011.04.007. 21624728 10.1016/j.socscimed.2011.04.007

[59] Dalmer N. Informing care: Mapping the social organization of families’ information work in an aging in place climate. Electronic Thesis and Dissertation Repository. 2018 [cited 25 Aug 2020]. Available: https://ir.lib.uwo.ca/etd/5948

[60] Zarzycki M, Seddon D, Bei E, Morrison V. Why do they care? A qualitative systematic review and meta-synthesis of personal and relational motivations for providing informal care. Health Psychol Rev. 2022. 10.1080/17437199.2022.2058581. 35383541 10.1080/17437199.2022.2058581

[61] Zarzycki M, Morrison V, Bei E, Seddon D. Cultural and societal motivations for being informal caregivers: a qualitative systematic review and meta-synthesis. Health Psychol Rev. 2022. 10.1080/17437199.2022.2032259. 35081864 10.1080/17437199.2022.2032259

[62] Ormandy P. Defining information need in health – assimilating complex theories derived from information science. Health Expect. 2011;14:92–104. 10.1111/J.1369-7625.2010.00598.X. 20550592 10.1111/j.1369-7625.2010.00598.x PMC5060560

[63] Bandura A. Self-efficacy: toward a unifying theory of behavioral change. Adv Behav Res Therapy. 1978;1:139–61. 10.1016/0146-6402(78)90002-4. 10.1037//0033-295x.84.2.191 847061

[64] Wilson TD. Information behaviour: an interdisciplinary perspective. Inf Process Manag. 1997;33:551–72. 10.1016/s0306-4573(97)00028-9.

[65] Wilson TD. Models in information behaviour research. J Doc. 1999;55:249–70. 10.1108/EUM0000000007145.

[66] Newbronner L, Chamberlain R, Borthwick R, Baxter M, Glendinning C. A Road Less Rocky-Supporting Carers of People with Dementia 1 A Road Less Rocky-Supporting Carers of People with Dementia. London; 2013. Available: https://carers.org/downloads/resources-pdfs/road-less-rocky/a-road-less-rocky--supporting-carers-of-people-with-dementia.pdf

[67] Hossain M, Crossland J, Stores R, Dewey A, Hakak Y. Awareness and understanding of dementia in South asians: a synthesis of qualitative evidence. https://doi.org/101177/1471301218800641. 2018;19: 1441–73. 10.1177/1471301218800641 10.1177/1471301218800641 30296834

[68] Killen A, Flynn D, De Brún A, O’Brien N, O’Brien J, Thomas AJ, et al. Support and information needs following a diagnosis of dementia with Lewy bodies. Int Psychogeriatr. 2016;28:495–501. 10.1017/S1041610215001362. 26328546 10.1017/S1041610215001362

[69] Ducharme F, Kergoat MJ, Coulombe R, Lvesque L, Antoine P, Pasquier F. Unmet support needs of early-onset dementia family caregivers: a mixed-design study. BMC Nurs. 2014;13. 10.1186/s12912-014-0049-3. 10.1186/s12912-014-0049-3 PMC4279790 25550685

