# Supplementary figures and images for: Explaining the unmet information needs of family carers of people with dementia: a theoretical model of information behaviour
Source: BMC Geriatr. 2025 Apr 3;25:219. doi: 10.1186/s12877-024-05626-6 (PMC11967026; doi:10.1186/s12877-024-05626-6)

## Slide 1
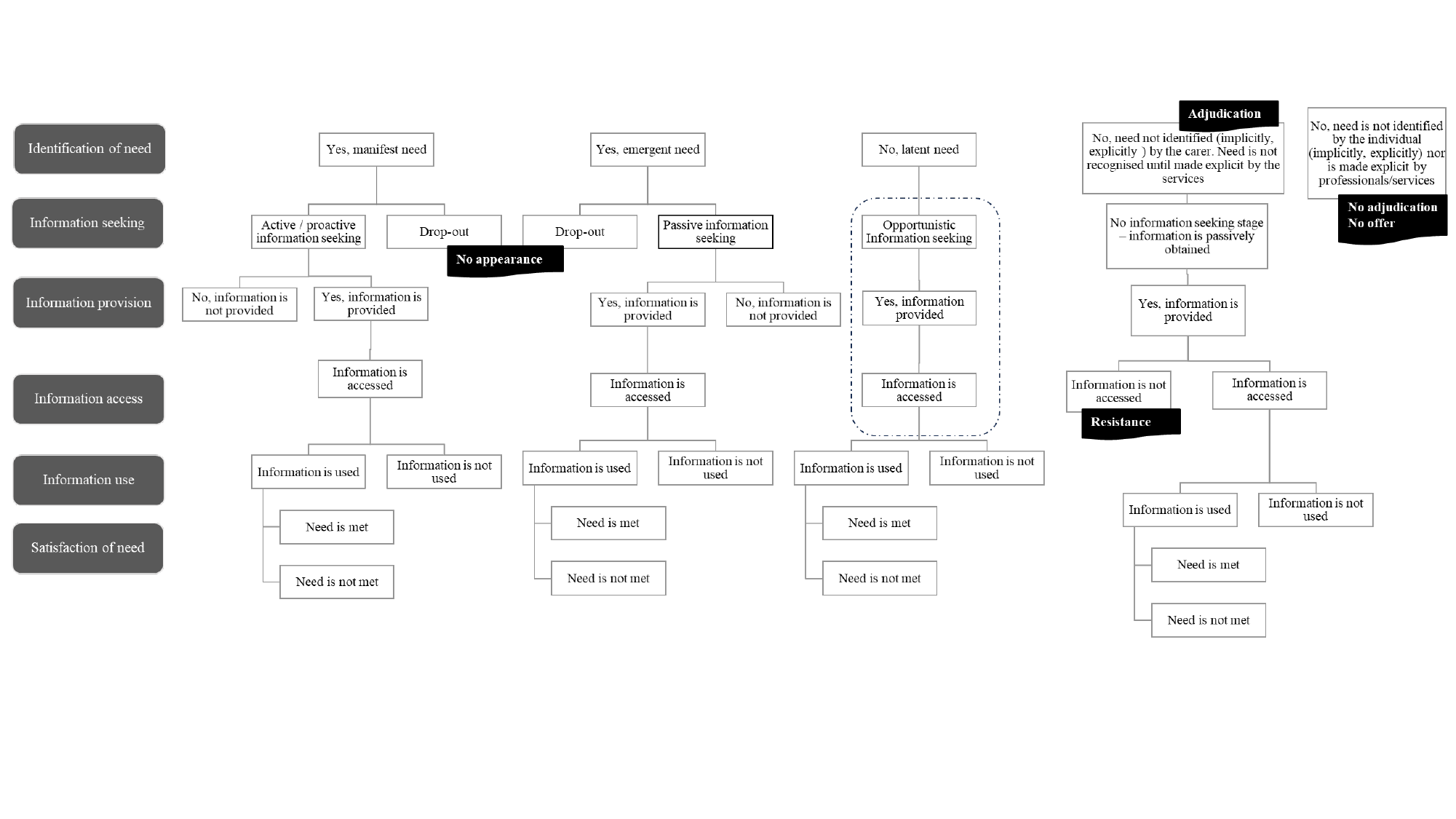

Supplement: Supplementary file 1 — Supplementary Material 1 [file 12877_2024_5626_MOESM1_ESM.pptx]
